# Correction: DFT/TDDFT insights into the chemistry, biochemistry and photophysics of copper coordination compounds

**DOI:** 10.1039/d0ra90110e

**Published:** 2020-10-16

**Authors:** Athanassios C. Tsipis

**Affiliations:** Laboratory of Inorganic and General Chemistry, Department of Chemistry, University of Ioannina 451 10 Ioannina Greece attsipis@uoi.gr

## Abstract

Correction for ‘DFT/TDDFT insights into the chemistry, biochemistry and photophysics of copper coordination compounds’ by Athanassios C. Tsipis, *RSC Adv.*, 2014, **4**, 32504–32529, DOI: 10.1039/C4RA04921G.

*RSC Advances* is issuing this correction to notify readers that there are portions of text overlap with a number of different sources in this review article. Although the sources have been cited in appropriate locations in this article, the text should have been rewritten to avoid the overlapping text.

## Supplementary Material

